# Prognostic value of admission NHHR for functional recovery in acute ischemic stroke

**DOI:** 10.3389/fendo.2026.1712473

**Published:** 2026-02-05

**Authors:** Xiangqi Kong, Xinyue Yuan, Haobo Wang, Mina Zhao, Wei Jing

**Affiliations:** Third Hospital of Shanxi Medical University, Shanxi Bethune Hospital, Shanxi Academy of Medical Sciences, Tongji Shanxi Hospital, Taiyuan, China

**Keywords:** AIS, functional outcome, NHHR, prognosis, risk stratification

## Abstract

**Background:**

Early risk stratification in acute ischemic stroke (AIS) is critical for guiding management and improving outcomes. The ratio of non–high-density lipoprotein to high-density lipoprotein cholesterol (NHHR) represents the equilibrium between pro-atherogenic and anti-atherogenic lipoproteins, potentially capturing both lipid metabolism and inflammatory status, but its prognostic value in AIS remains unclear.

**Methods:**

We retrospectively analyzed 812 first-ever AIS patients. Admission NHHR was calculated from routine lipid panels. The study’s main outcome was 90-day functional status, determined using the modified Rankin Scale. Associations with NHHR were examined through multivariable logistic regression, quartile analyses, restricted cubic spline (RCS) modeling, and subgroup analyses.

**Results:**

Among 812 patients, 255 (31.4%) had unfavorable outcomes. Elevated NHHR showed an independent correlation with unfavorable 90-day functional outcomes (adjusted OR per unit increase, 1.233; 95% CI, 1.058–1.439; P = 0.0076). RCS analysis demonstrated a gradual rise in risk with increasing NHHR (P for trend = 0.043; P for nonlinearity = 0.584). Subgroup analyses showed consistent associations across sex, stroke severity, smoking, drinking, hypertension, and diabetes.

**Conclusion:**

Admission NHHR is a simple, inexpensive, and robust predictor of 90-day unfavorable outcomes in AIS. It provides a novel integrated lipid–inflammation marker for early risk stratification, warranting further validation in prospective studies.

## 1 Introduction

Acute ischemic stroke (AIS) remains a major global cause of mortality and prolonged disability, exerting significant impact on patients, families, and healthcare resources (1). Reperfusion strategies, including intravenous thrombolysis and endovascular thrombectomy, have significantly improved rates of early vascular recanalization (2). Accordingly, in the acute phase of AIS, the identification of reliable and accessible biomarkers for early risk stratification, individualized treatment, and improved long-term outcomes has emerged as a major priority in stroke research (3).

Atherosclerosis represents the predominant pathological basis of AIS, with lipid metabolism disorders playing a central role in its development (4). Traditionally, low-density lipoprotein cholesterol (LDL) remains a central marker for cardiovascular risk assessment and treatment (5). However, emerging findings show that non–high-density lipoprotein cholesterol (non-HDL), which encompasses all atherogenic apolipoprotein-containing lipoproteins, provides superior prognostic value for cardiovascular risk compared with LDL (6). In parallel, HDL has been recognized for its protective roles, including anti-atherosclerotic, antioxidant, and anti-inflammatory effects (7). Consequently, the non-HDL to HDL ratio (NHHR), as an integrated marker, may better capture the equilibrium between atherogenic and protective lipoproteins.

Compelling evidence has been accumulated regarding the prognostic role of NHHR in cardiovascular disease. Several large-scale epidemiologic investigations suggest that NHHR is a powerful predictor of future cardiovascular outcomes in high-risk populations, including individuals with hypertension (8) or diabetes (9), with predictive performance superior to conventional lipid parameters including LDL and total cholesterol (TC). In coronary artery disease, NHHR has been shown to correlate significantly with the severity of coronary lesions (10) and to serve as an independent predictor of non-culprit lesion progression after percutaneous coronary intervention (11). The prognostic utility of NHHR is based on its capacity to simultaneously capture the overall burden of atherogenic cholesterol (non-HDL) and the protective level of anti-atherogenic cholesterol (HDL), thereby providing a more comprehensive risk profile of atherosclerosis.

It is noteworthy that atherosclerosis is fundamentally a chronic inflammatory disease (12), while inflammatory responses also play a pivotal role in secondary brain injury following AIS (13). NHHR provides a unique link between lipid metabolic homeostasis and the inflammatory milieu. Specifically, non-HDL levels have been positively associated with chronic inflammatory states (14), whereas HDL has been linked to anti-inflammatory effects (7). Thus, NHHR may serve as a robust composite predictor that simultaneously reflects both the underlying atherosclerotic burden and the acute inflammatory response in patients with stroke.

In contrast to the cardiovascular field, research on NHHR in cerebrovascular disease—particularly in the prognostic assessment of AIS—remains relatively limited. Preliminary evidence has suggested that elevated NHHR is associated with an increased risk of stroke in U.S. populations (15). However, its relationship with long-term functional outcomes in AIS, such as 90-day modified Rankin Scale (mRS) scores, has not been clearly established, and high-quality evidence from large-scale cohorts is still lacking.

We investigated the prognostic relevance of admission NHHR for 90-day outcomes (mRS >2) in AIS. We hypothesized that elevated NHHR could function as an independent risk factor for poor prognosis at 90 days. This study’s findings may help establish the prognostic value of NHHR in stroke and provide a novel, practical, and integrative biomarker that captures both lipid metabolism and inflammatory status for early risk stratification in AIS.

## 2 Materials and methods

### 2.1 Study population

We conducted a retrospective analysis of patients with acute ischemic stroke (AIS) admitted to the Department of Neurology, Shanxi Bethune Hospital, between October 2022 and September 2024. AIS was defined according to World Health Organization criteria, and all diagnoses were confirmed by magnetic resonance imaging (MRI) or computed tomography (CT).

Patients were included if they (1) were diagnosed with AIS by CT or MRI within 72 hours of symptom onset, in accordance with the *2018 Chinese Guidelines for the Diagnosis and Treatment of Acute Ischemic Stroke*; (2) had a first-ever stroke without a history of severe neurological deficits; (3) were aged >18 years; and (4) had complete clinical data and provided informed consent.

Exclusion criteria were: (1) admission >72 hours after stroke onset; (2) other vascular causes of ischemia or cerebral venous thrombosis; (3) intracranial tumors, tumor-related stroke, or intracranial infections; (4) neurological deficits from non-neurological conditions, including malignancy, severe pneumonia, hematologic disorders, or severe cardiac, hepatic, renal, or bleeding disorders; (5) prior stroke with ongoing antiplatelet or anticoagulant therapy; (6) receipt of intravenous thrombolysis or endovascular therapy; (7) chronic inflammatory disease; (8) incomplete clinical or laboratory data; and (9) loss to follow-up.

**Figure 1:**
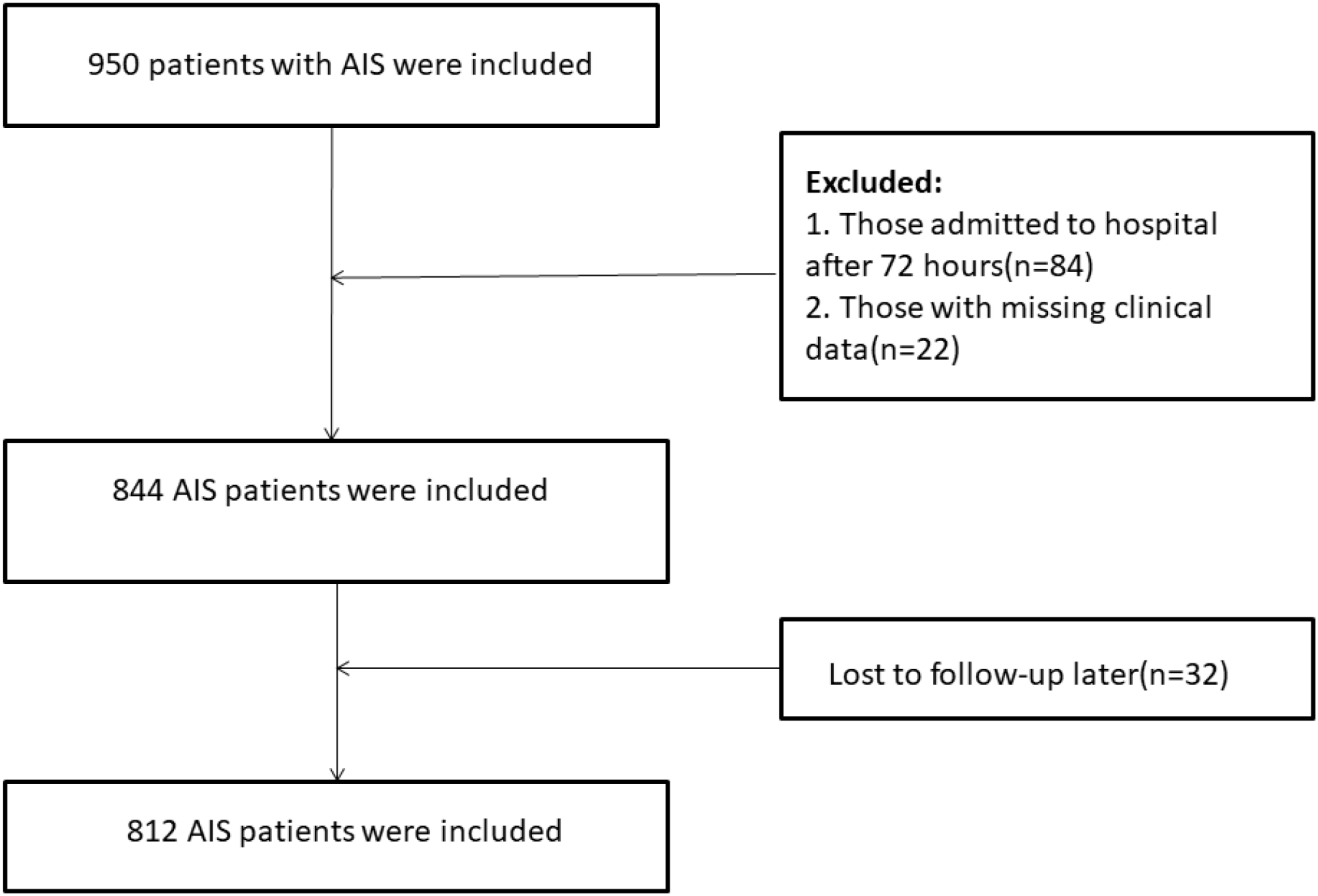
Flowchart of participant selection.

A total of 812 patients met the eligibility criteria and were included in the final analysis (**Figure 1**).

### 2.2 Data collection

Baseline demographic and clinical variables were recorded, including age, sex, hypertension, diabetes mellitus, coronary artery disease, smoking, alcohol use, and NIHSS score. Venous blood (2 mL) was collected in EDTA-K2 tubes for measurement of leukocyte, neutrophil, monocyte, lymphocyte, and platelet counts using an automated hematology analyzer (Beckman DXH800). An additional 5 mL of blood was drawn into plain tubes, centrifuged, and analyzed for uric acid, total cholesterol, triglycerides, HDL-C, LDL-C, homocysteine, and myeloperoxidase. Plasma D-dimer was measured from citrated samples (1:9 sodium citrate) using the ACL-TOP 750 system (Werfen). Serum lipid parameters, including total cholesterol and high-density lipoprotein cholesterol, were measured in mmol/L. All blood samples were collected under fasting conditions at admission according to routine clinical protocols. The NHHR ratio was calculated as (TC − HDL)/HDL.

### 2.3 Outcome assessment

The primary outcome was functional status at 90 days, assessed using the modified Rankin Scale (mRS). A favorable outcome was defined as mRS ≤2, and an unfavorable outcome as mRS >2. Stroke severity at admission was evaluated with the National Institutes of Health Stroke Scale (NIHSS), with scores <4 indicating mild stroke and scores ≥4 indicating moderate-to-severe stroke.

### 2.4 Statistical analysis

Baseline characteristics were compared according to 90-day functional outcome. Normality of continuous variables was assessed with the Shapiro–Wilk test. Normally distributed variables were expressed as mean ± standard deviation and compared using the Student’s *t*-test; non-normally distributed variables were presented as median (25th–75th percentile) and compared using the Mann–Whitney *U* test. Categorical variables were expressed as frequencies and percentages, and differences were examined with the chi-square test.

Multivariable logistic regression was performed to evaluate the association between NHHR and unfavorable outcome. Covariates included in the multivariable models were selected based on clinical relevance, evidence from previous literature, and results of univariable regression analysis. Variables showing a significant association with 90-day functional outcomes in univariable analysis (P < 0.05) were considered for inclusion in the multivariable models. Three models were constructed: Model 1, unadjusted; Model 2, adjusted for age and sex; and Model 3, further adjusted for NIHSS score, smoking, drinking, CAD, D-dimer, and MPO. Diabetes mellitus was examined but did not reach statistical significance and was therefore not included in subsequent multivariable analyses. Multicollinearity among covariates was assessed using variance inflation factors (VIFs) at different analytic stages. At the variable selection stage, substantial collinearity was observed when TC, HDL, and NHHR were simultaneously included, reflecting their structural dependence (**Supplementary Table S2**). Therefore, TC and HDL were excluded from subsequent models. In the final multivariable Model 3, all retained variables, including NHHR, demonstrated low VIF values, indicating no clinically relevant multicollinearity (**Supplementary Table S3**).

Sensitivity analyses included modeling NHHR in quartiles with trend tests and using restricted cubic splines with four knots to explore potential nonlinear associations. Restricted cubic spline analyses were performed to explore the potential nonlinear association between admission NHHR and 90-day unfavorable functional outcome. Four knots were placed at the 5th, 35th, 65th, and 95th percentiles of NHHR, and the median NHHR value was used as the reference point. Subgroup and interaction analyses were conducted by stroke severity, sex, smoking, drinking, hypertension, and diabetes status. Each subgroup analysis was adjusted for the same covariates as Model 3, except for the stratification variable itself.

All statistical analyses were performed using R software (version 4.4.2) and DecisionLinnc 1.0. A two-sided *p* value <0.05 was considered statistically significant.

## 3 Results

### 3.1 Baseline characteristics

A total of 812 patients were included, of whom 557 had favorable outcomes and 255 had unfavorable outcomes (**Table 1**). Significant differences between groups were observed in sex, smoking, drinking, coronary artery disease (CAD), NIHSS score, D-dimer, uric acid (UA), myeloperoxidase (MPO), high-density lipoprotein cholesterol (HDL), homocysteine (Hcy), and NHHR (all *P* < 0.05). Specifically, patients with unfavorable outcomes were more likely to be male, smokers, and drinkers, and had higher NIHSS scores. They also exhibited elevated levels of D-dimer, UA, MPO, and Hcy, along with reduced HDL levels. In addition, NHHR was significantly higher in the unfavorable outcome group.

**Table 1:** Baselines characteristics of participants.

Characteristic	Overall N = 812^1^	Favorable outcome N = 557^1^	Unfavorable outcome N = 255^1^	p-value^2^
Gender				<0.001
Female	250 (31%)	200 (36%)	50 (20%)	
Male	562 (69%)	357 (64%)	205 (80%)	
Smoking				0.002
No	359 (44%)	267 (48%)	92 (36%)	
Yes	453 (56%)	290 (52%)	163 (64%)	
Drinking				0.009
No	437 (54%)	317 (57%)	120 (47%)	
Yes	375 (46%)	240 (43%)	135 (53%)	
HTN				0.148
No	326 (40%)	233 (42%)	93 (36%)	
Yes	486 (60%)	324 (58%)	162 (64%)	
DM				0.060
No	562 (69%)	397 (71%)	165 (65%)	
Yes	250 (31%)	160 (29%)	90 (35%)	
CAD				0.001
No	750 (92%)	503 (90%)	247 (97%)	
Yes	62 (7.6%)	54 (9.7%)	8 (3.1%)	
NIHSS	2.00 (1.00, 3.00)	2.00 (1.00, 3.00)	3.00 (2.00, 5.00)	<0.001
Age(years)	63.00 (55.00, 70.00)	63.00 (54.00, 70.00)	63.00 (56.00, 70.00)	0.854
WBC(×10^9/L)	6.40 (5.40, 7.70)	6.40 (5.30, 7.70)	6.30 (5.50, 7.80)	0.459
Lym(×10^9/L)	2.13 (1.57, 2.90)	2.11 (1.55, 2.90)	2.21 (1.58, 2.91)	0.727
Mon(×10^9/L)	0.52 (0.41, 0.64)	0.52 (0.40, 0.64)	0.53 (0.43, 0.64)	0.316
Neu(×10^9/L)	5.20 (3.49, 6.43)	5.26 (3.61, 6.41)	4.88 (3.26, 6.61)	0.350
PLT(×10^9/L)	218.98 (182.00, 265.83)	218.08 (182.00, 264.48)	219.00 (182.12, 270.28)	0.645
D-dimer(ng/mL)	115.50 (70.00, 212.00)	103.00 (65.00, 189.00)	155.00 (88.00, 266.00)	<0.001
UA(mmol/L)	296.00 (240.70, 368.80)	289.70 (237.90, 357.90)	305.50 (253.40, 386.20)	0.017
TG(mmol/L)	1.44 (1.11, 1.83)	1.45 (1.11, 1.89)	1.44 (1.13, 1.73)	0.779
TC(mmol/L)	4.15 (3.45, 4.86)	4.16 (3.53, 4.88)	4.08 (3.34, 4.76)	0.055
LDL(mmol/L)	2.63 (2.19, 3.12)	2.61 (2.19, 3.18)	2.67 (2.17, 3.07)	0.809
MPO(ng/mL)	137.30 (86.85, 206.75)	119.10 (79.20, 189.60)	173.20 (106.40, 238.50)	<0.001
HDL(mmol/L)	0.97 (0.84, 1.15)	1.01 (0.88, 1.21)	0.87 (0.77, 1.05)	<0.001
Hcy(umol/L)	16.00 (12.50, 24.50)	15.60 (12.50, 23.40)	17.70 (13.20, 27.10)	0.004
NHHR	3.14 (2.49, 3.97)	3.04 (2.37, 3.84)	3.48 (2.72, 4.31)	<0.001

1n (%); Median (Q1, Q3)

2Pearson’s Chi-squared test; Wilcoxon rank sum test.

Note: HTN: hypertension; DM: diabetes mellitus; CAD: coronary artery disease; WBC: white blood cell; Lym: lymphocyte; Mon: monocyte; Neu: neutrophil; PLT: platelet counts; UA: uric acid; TG: triglycerides; TC:total cholesterol; LDL: low-density lipoprotein; MPO: myeloperoxidase; HDL: high-density lipoprotein; Hcy: homocysteine; NHHR: Non-high-density lipoprotein/high-density lipoprotein

### 3.2 Association of NHHR with 90-day functional outcomes

Univariable regression analysis (**Supplementary Table 1**) indicated that sex, smoking, drinking, CAD, NIHSS score, D-dimer, TC, MPO, HDL, and NHHR were all associated with 90-day functional outcomes in patients with AIS. Multivariable logistic regression (**Table 2**) further demonstrated a significant positive association between NHHR and unfavorable outcomes.

**Table 2:** Multivariable logistic regression analysis of the association between NHHR and 90-day functional prognosis of AIS.

Exposures	Model 1 OR(95%CI),*P*-value	Model 2 OR(95%CI),*P*-value	Model 3 OR(95% CI), *P*-value
NHHR	1.4748(1.2905, 1.6914),<0.0001	1.4550(1.2714, 1.6710), <0.0001	1.2326(1.0579, 1.4387),0.0076
Quartiles			
Q1	Reference	Reference	Reference
Q2	1.3429(0.8530, 2.1240),0.2043	1.3358(0.8432, 2.1259),0.2188	1.1005(0.6552, 1.8538),0.7176
Q3	1.7409(1.1193, 2.7281),0.0145	1.6674(1.0653, 2.6284),0.0262	1.4216(0.8566, 2.3729),0.1751
Q4	2.8781(1.8746, 4.4693), <0.0001	2.7625(1.7881, 4.3150), <0.0001	1.5683(0.9439, 2.6197),0.0834
P for trend	<0.0001	<0.0001	0.0477

Model 1 = no covariates were adjusted.

Model 2 = Model 1 + age, gender were adjusted.

Model3 = Model2+NIHSS,smoking,drinking, CAD, D-dmier, MPO were adjusted.

When analyzed as a continuous variable, higher NHHR was independently associated with increased risk of poor outcome. In Model 1 (unadjusted), each unit increase in NHHR was associated with a 47.5% higher risk (OR = 1.4748; 95% CI, 1.2905–1.6914; *P* < 0.0001). After adjusting for age and sex (Model 2), the association remained robust (OR = 1.4550; 95% CI, 1.2714–1.6710; *P* < 0.0001). With further adjustment for NIHSS score, smoking, drinking, CAD, diabetes, and MPO (Model 3), NHHR remained significantly associated with unfavorable outcome (OR = 1.2326; 95% CI, 1.0579–1.4387; *P* = 0.0076).

Quartile-based analysis showed a dose–response relationship between NHHR and unfavorable outcomes. In both unadjusted and demographically adjusted models, patients in the highest quartile (Q4) had a markedly increased risk compared with those in the lowest quartile (Q1) (Model 1: OR = 2.8781; 95% CI, 1.8746–4.4693; Model 2: OR = 2.7625; 95% CI, 1.7881–4.3150; both *P* < 0.0001). Although the association attenuated and lost statistical significance after full adjustment (Model 3: OR = 1.5683; 95% CI, 0.9439–2.6197; *P* = 0.0834), the test for trend still supported a dose–response relationship (*P* for trend = 0.0477).

### 3.3 Exploration of nonlinear associations

**Figure 2:**
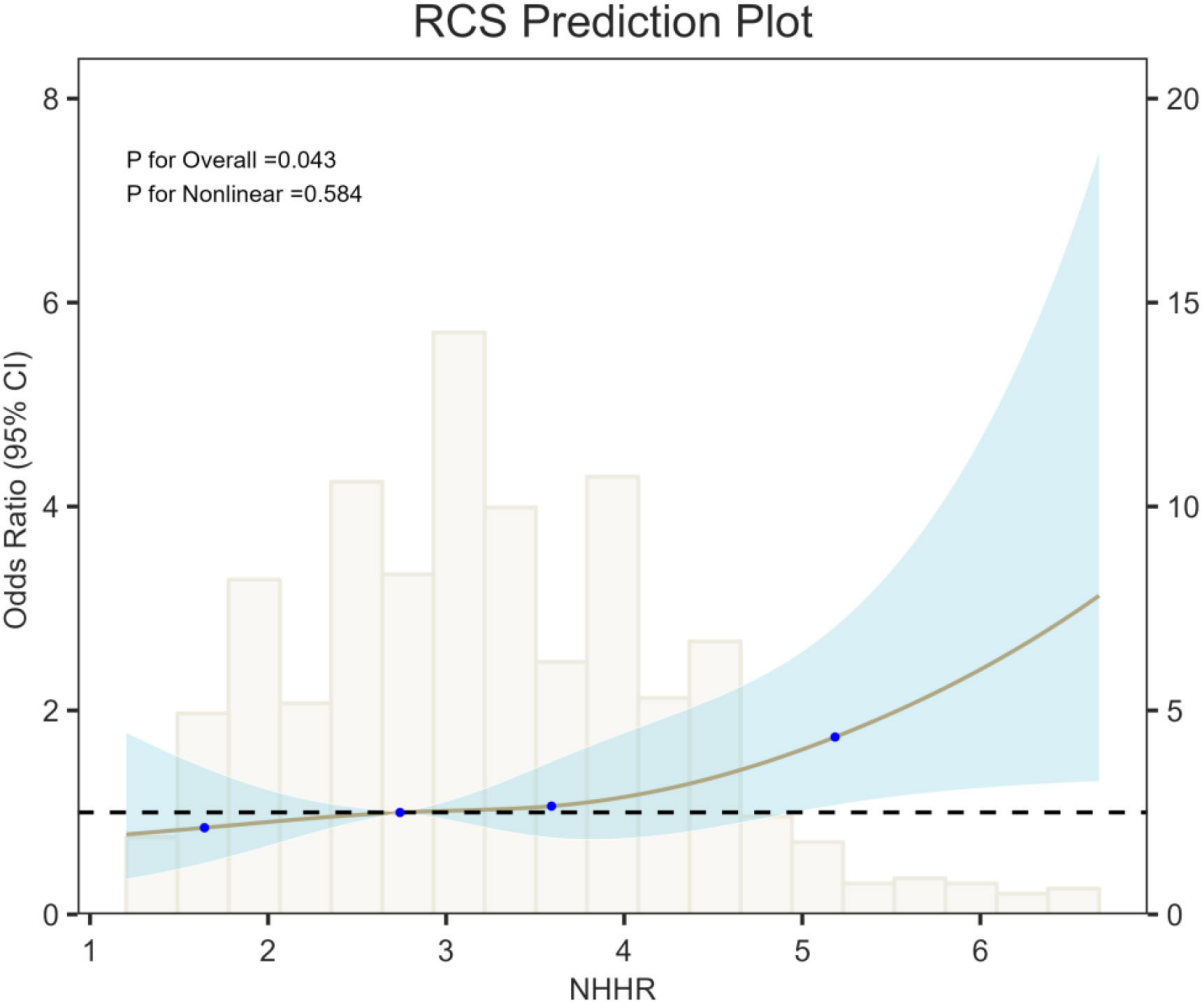
RCS showing the association between NHHR and 90-day functional prognosis of AIS.

RCS analysis (**Figure 2**) showed that the risk of unfavorable 90-day functional outcome increased progressively with higher NHHR, demonstrating a significant positive association (*P* for overall trend = 0.043). However, the test for nonlinearity was not significant (*P* = 0.584), suggesting no evidence of a nonlinear relationship between NHHR and 90-day outcomes. Clinically, the spline curve indicates a steady increase in outcome risk across the observed NHHR range, without an apparent threshold or inflection point.

### 3.4 Subgroup analysis

**Figure 3:**
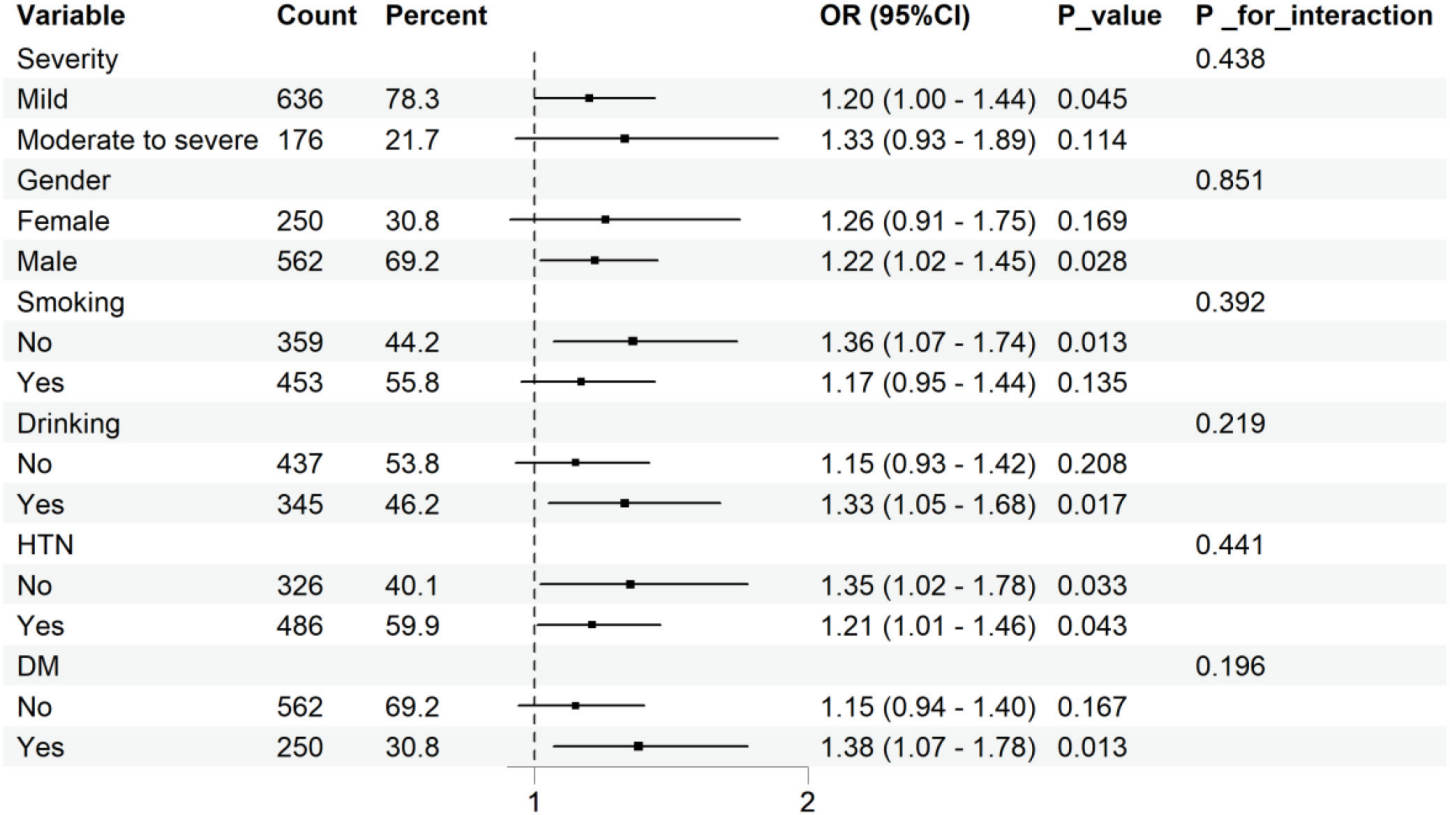
Subgroup analysis for the association between NHHR and 90-day functional prognosis of AIS.

Subgroup analyses were conducted according to stroke severity, sex, smoking, drinking, hypertension, and diabetes status. No significant interactions were observed between NHHR and subgroup variables, as shown in **Figure 3** (*P* for interaction >0.05).

## 4 Discussion

In 812 patients with first-ever AIS who did not receive reperfusion therapy, we demonstrated that admission NHHR was independently and continuously associated with unfavorable 90-day functional outcomes (mRS > 2) in a robust dose–response manner. After adjustment for age, sex, stroke severity, comorbidities, and inflammatory markers, every unit increase in NHHR was linked to a 23.3% higher risk of poor outcome. Although the association between the highest NHHR quartile and unfavorable outcome was attenuated after full adjustment, analyses treating NHHR as a continuous variable remained statistically significant. Restricted cubic spline analysis did not reveal significant nonlinearity, and trend tests across NHHR quartiles supported an overall exposure–response relationship, indicating that the risk of unfavorable outcome increased progressively with higher NHHR levels. Subgroup analyses showed no significant interactions, indicating that this association was consistent across different clinical populations.

Our findings are highly consistent with prior studies of NHHR in the cardiovascular field, further supporting its potential as a universal prognostic marker for atherosclerosis-related diseases. Among individuals with coronary artery disease, NHHR has been shown to predict incident CAD (16) and serves as an independent predictor of adverse cardiovascular events after percutaneous coronary intervention (11);Similarly, in high-risk individuals including patients with hypertension (17),diabetes (18), and congestive heart failure (19), NHHR effectively predicts endpoints including cardiovascular and mortality. Our study extends the application of NHHR to the prognosis of AIS patients and demonstrates that its association with unfavorable 90-day outcomes—characterized by independence and a dose–response relationship—mirrors findings in cardiovascular disease research. These results indicate that NHHR could be a general prognostic indicator across the cardiovascular disease spectrum by reflecting systemic atherosclerosis and inflammatory status.

The core finding of our research—that NHHR is an independent predictor of AIS prognosis—aligns closely with the dual “lipid–inflammation” mechanism of atherosclerosis. NHHR integrates the “total atherogenic lipoprotein burden” (Non-HDL) with the “protective capacity of antioxidant, anti-inflammatory, and cholesterol efflux functions” (HDL) into a single ratio. A high NHHR reflects an imbalance characterized by “high aggressiveness and low defense”: elevated Non-HDL indicates not only a broader and more unstable baseline atherosclerotic plaque burden but also a pro-thrombotic blood environment (20, 21). Conversely, reduced HDL diminishes cholesterol efflux, antioxidant activity, and endothelial protective functions (22, 23). Clinical studies have shown that individuals with high NHHR exhibit increased arterial stiffness (24) and are more likely to have intracranial atherosclerotic stenosis (25). Beyond promoting atherosclerosis, NHHR may exacerbate secondary brain injury through synergistic inflammatory mechanisms. Elevated Non-HDL can activate signaling pathways such as TLR4/NF-κB to promote the release of inflammatory mediators, amplifying ischemic penumbra inflammation (26, 27),whereas HDL exerts anti-inflammatory effects by inhibiting immune cell activation and facilitating clearance of inflammatory mediators (28, 29). In our cohort, NHHR was concomitantly elevated with MPO and D-dimer, suggesting that it may aggravate secondary brain injury via the “oxidative stress–microthrombosis axis.” Notably, even after adjustment for the acute inflammatory marker MPO in Model 3, NHHR remained a significant predictor, indicating that it primarily reflects the patient’s pre-existing chronic atherosclerotic risk rather than the acute inflammatory response alone.

Furthermore, our findings should be interpreted in the context of “residual cardiovascular risk,” a concept characterized by persistent inflammation and elevated remnant cholesterol levels despite seemingly controlled LDL or non-HDL (30). Since Non-HDL (the numerator of NHHR) includes remnant cholesterol, NHHR may serve as a simple and accessible marker that integrates the atherogenic burden of remnant lipids with the anti-inflammatory/protective capacity of HDL. This perspective suggests that NHHR could help identify patients with “residual risk” who might benefit from more targeted therapeutic strategies beyond traditional LDL-lowering approaches.

Current research on NHHR in cerebrovascular disease remains limited. To date, just one piece of research has suggested that higher NHHR is associated with stroke occurrence in U.S. adults (15). However, its relationship with long-term functional outcomes in AIS patients—such as 90-day mRS—has not been established, and analyses focusing on patients who did not receive reperfusion therapy are lacking. This study addresses these gaps in two ways. First, using 90-day mRS—a globally recognized gold standard for stroke functional outcomes—as the endpoint, we report for the first time that admission NHHR is directly linked to long-term functional recovery in AIS patients. This association remained independent after adjusting for NIHSS score, a key indicator of stroke severity, indicating that NHHR not only reflects baseline pathology but also provides incremental prognostic information. Second, we excluded patients who received intravenous thrombolysis or endovascular therapy. Although such interventions are common in clinical AIS populations, particularly in primary hospitals, the prognosis of patients without reperfusion therapy largely depends on their intrinsic pathophysiological status. Our findings confirm that NHHR can serve as a simple and practical risk stratification tool for this patient population.

The findings of this study have potential clinical translational value. NHHR can be calculated from routine admission blood tests (complete lipid profile) without additional cost, and results are immediately available, making it well suited for rapid implementation in emergency and neurology settings. Early identification of patients with elevated NHHR may enable clinicians to provide more intensive monitoring, initiate enhanced secondary prevention strategies earlier, or potentially enroll high-risk individuals in future trials of personalized interventions, such as intensified lipid-lowering or anti-inflammatory therapies, ultimately improving long-term functional recovery.

This study is subject to limitations, including its single-center retrospective design. Although multivariable models adjusted for potential confounders, unmeasured confounding may remain, and the findings should be interpreted cautiously. Second, only a single NHHR measurement at admission was analyzed, precluding assessment of temporal changes and their relationship with outcomes; future studies could explore the value of serial monitoring. Third, by excluding certain critically ill patients and those who received reperfusion therapy, participants in this study were relatively “homogeneous,” which could limit the applicability of the findings to broader AIS populations, particularly patients undergoing endovascular treatment. Future multicenter studies incorporating patients undergoing intravenous thrombolysis (IVT) or endovascular therapy (EVT) are needed to determine whether the prognostic value of NHHR extends to reperfusion-treated populations. In addition, information on statin use prior to admission was not consistently available in this retrospective dataset and therefore could not be included in the multivariable analyses, which may have introduced residual confounding. Finally, the precise molecular mechanisms through which NHHR influences prognosis remain to be elucidated in basic research.

## 5 Conclusion

This study demonstrates that admission NHHR is an independent prognostic factor of unfavorable functional outcomes in AIS. As a simple, inexpensive, and robust marker, NHHR provides a novel integrated lipid–inflammation parameter for early risk stratification in clinical practice. Prospective research is warranted to further establish causality and assess its utility in targeted treatment.

## Data Availability

The original contributions presented in the study are included in the article/**Supplementary Material**. Further inquiries can be directed to the corresponding author.
