# Supplementary material for: Prognostic value of admission NHHR for functional recovery in acute ischemic stroke
Source: Front Endocrinol (Lausanne). 2026 Feb 5;17:1712473. doi: 10.3389/fendo.2026.1712473 (PMC12916411; doi:10.3389/fendo.2026.1712473)
Supplement: Supplementary file 1 [file Table1.docx]

Catalogue

[Table S1 Univariate logistic regression analysis 2](#_Toc219654788)

[Table S2 Variance Inflation Factor (VIF) Analysis at the Variable Selection Stage for Variables with Significant Univariate Associations and Demographic Characteristics 3](#_Toc219654789)

[Table S3 Variance Inflation Factor (VIF) Analysis for the Final Multivariable Model 4](#_Toc219654790)

## Table S1 Univariate logistic regression analysis

| Variable Name | OR（95%CI） | P value |
| --- | --- | --- |
| NIHSS | 1.8101 （1.6329—2.0194） | 0.0000 |
| age | 1.0017 （0.9880—1.0157） | 0.8058 |
| WBC | 1.0598 （0.9929—1.1308） | 0.0790 |
| Lym | 0.9894 （0.8535—1.1443） | 0.8869 |
| Mon | 1.0521 （0.5606—1.8484） | 0.8550 |
| Neu | 0.9889 （0.9182—1.0628） | 0.7639 |
| PLT | 1.0007 （0.9986—1.0029） | 0.5025 |
| D-dimer | 1.0006 （1.0001—1.0011） | 0.0162 |
| UA | 1.0015 （0.9999—1.0030） | 0.0591 |
| TG | 1.0593 （0.8538—1.3058） | 0.5925 |
| TC | 0.8343 （0.7184—0.9659） | 0.0163 |
| NHHR | 1.4748 （1.2905—1.6914） | 0.0000 |
| LDL | 0.9944 （0.8169—1.2089） | 0.9550 |
| MPO | 1.0055 （1.0039—1.0071） | 0.0000 |
| HDL | 0.0569 （0.0262—0.1183） | 0.0000 |
| Hcy | 1.0083 （0.9980—1.0186） | 0.1092 |
| gender | 2.2969 （1.6221—3.2988） | 0.0000 |
| smoking | 1.6312 （1.2048—2.2179） | 0.0017 |
| drinking | 1.4859 （1.1039—2.0027） | 0.0091 |
| HTN | 1.2527 （0.9243—1.7039） | 0.1484 |
| DM | 1.3534 （0.9854—1.8538） | 0.0603 |
| CAD | 0.3017 （0.1310—0.6085） | 0.0019 |

## Table S2 Variance Inflation Factor (VIF) Analysis at the Variable Selection Stage for Variables with Significant Univariate Associations and Demographic Characteristics

| Variable Name | VIF |
| --- | --- |
| NIHSS | 1.08 |
| Age | 1.15 |
| smoking | 1.64 |
| Drinking | 1.49 |
| gender | 1.43 |
| TC | 10.73 |
| NHHR | 12.70 |
| CAD | 1.10 |
| D-dimer | 1.10 |
| MPO | 1.10 |
| HDL | 8.79 |

## Table S3 Variance Inflation Factor (VIF) Analysis for the Final Multivariable Model

| Variable Name | VIF |
| --- | --- |
| NIHSS | 1.08 |
| Age | 1.13 |
| smoking | 1.64 |
| Drinking | 1.51 |
| gender | 1.44 |
| NHHR | 1.03 |
| CAD | 1.10 |
| D-dimer | 1.10 |
| MPO | 1.10 |
